# Two Human ARFGAPs Associated with COP-I-Coated Vesicles

**DOI:** 10.1111/j.1600-0854.2007.00631.x

**Published:** 2007-08-29

**Authors:** Gabriella Frigerio, Neil Grimsey, Martin Dale, Irina Majoul, Rainer Duden

**Affiliations:** 1 Department of Clinical Biochemistry, Cambridge Institute for Medical Research, University of Cambridge Hills Road, Cambridge CB2 2XY, United Kingdom; 2 European Bioinformatics Institute, Wellcome Trust Genome Campus Hinxton, Cambridge CB10 1SD, United Kingdom; 3 Centre for Biomedical Sciences, School of Biological Sciences, Royal Holloway University of London Egham TW20 0EX, United Kingdom

**Keywords:** ARF, ARFGAP, coated vesicles, coatomer, COP I, Golgi

## Abstract

ADP-ribosylation factors (ARFs) are critical regulators of vesicular trafficking pathways and act at multiple intracellular sites. ADP-ribosylation factor-GTPase-activating proteins (ARFGAPs) are proposed to contribute to site-specific regulation. In yeast, two distinct proteins, Glo3p and Gcs1p, together provide overlapping, essential ARFGAP function required for coat protein (COP)-I-dependent trafficking. In mammalian cells, only the Gcs1p orthologue, named ARFGAP1, has been characterized in detail. However, Glo3p is known to make the stronger contribution to COP I traffic in yeast. Here, based on a conserved signature motif close to the carboxy terminus, we identify ARFGAP2 and ARFGAP3 as the human orthologues of yeast Glo3p. By immunofluorescence (IF), ARFGAP2 and ARFGAP3 are closely colocalized with coatomer subunits in NRK cells in the Golgi complex and peripheral punctate structures. In contrast to ARFGAP1, both ARFGAP2 and ARFGAP3 are associated with COP-I-coated vesicles generated from Golgi membranes in the presence of GTP-γ-S *in vitro*. ARFGAP2 lacking its zinc finger domain directly binds to coatomer. Expression of this truncated mutant (ΔN-ARFGAP2) inhibits COP-I-dependent Golgi-to-endoplasmic reticulum transport of cholera toxin (CTX-K63) *in vivo.* Silencing of ARFGAP1 or a combination of ARFGAP2 and ARFGAP3 in HeLa cells does not decrease cell viability. However, silencing all three ARFGAPs causes cell death. Our data provide strong evidence that ARFGAP2 and ARFGAP3 function in COP I traffic.

Cytoplasmic coat proteins govern the transport of proteins between membrane-bound compartments of the secretory and endocytic pathways by shaping the membrane of the donor organelle into vesicular or tubular transport carriers and selecting appropriate cargo into them (1–3). The well-characterized minimal machinery to form coat protein (COP)-I-coated vesicles from Golgi membranes comprises coatomer, a stable heptameric protein complex comprising α-, β-, β’-, γ-, δ-, ɛ- and ζ-COP, and the small ras-like GTPase ADP-ribosylation factor (ARF) in its GTP-bound form (4,5). The GTP/GDP cycle of ARF proteins is regulated by guanine nucleotide exchange factors and GTPase-activating proteins (GAPs) (6). ARF inactivation by GTP hydrolysis relies on stimulation of a low intrinsic GTPase activity by ARF-GTPase-activating proteins (ARFGAPs). ARFGAPs form a large family of proteins that share a conserved catalytic domain of approximately 70 residues that includes a zinc finger motif, but they differ in their non-catalytic domains (for review see 6,7). In this pathway, ARFGAP activity triggers uncoating of COP-I-coated vesicles through GTP hydrolysis on ARF, which renders both ARF and coatomer cytosolic (4). Interestingly, regulated GTP hydrolysis on ARF is required not only for uncoating but also for cargo selection into COP I vesicles because vesicles produced in the presence of the nonhydrolysable GTP analogue, GTP-γ-S, are depleted of protein cargo (8–10).

There is good evidence that a single ARF species may have multiple roles and been in different locations in the cell (6,11). It is generally thought that ARFGAPs with restricted cellular localizations will contribute to differential regulation of ARF to enable it to function in this way. In yeast, two ARFGAPs, Gcs1p and Glo3p, provide an overlapping essential function in the COP-I-mediated Golgi-to-endoplasmic reticulum (ER) transport (12,13). Several lines of evidence suggest that Glo3p makes the stronger contribution to COP-I-mediated trafficking. glo3Δ mutants display a much stronger retrograde Golgi-to-ER transport defect than gcs1Δ mutants (12). Glo3p but not Gcs1p is associated with yeast COP-I-coated vesicles formed *in vitro*(14). A mutant in *GLO3*, named *ret4-1*(13), was isolated based on a genetic selection for mutants with defects in dilysine motif-dependent Golgi-to-ER retrieval that had previously identified several coatomer subunits (15). Lastly, Nakano’s laboratory has recently shown that GLO3 but not GCS1 can suppress the temperature-sensitive (ts)-growth defect of the *arf1-16* and *arf1-17* mutants, which display strong defects in retrograde trafficking for several cargo proteins (16). These data provide compelling evidence that Glo3p in yeast has a critical function in COP-I-dependent traffic that cannot be complemented by Gcs1p. We have previously shown that Glo3p but not Gcs1p can interact with yeast coatomer *in vitro*(17,18). Using the two-hybrid system and *in vitro* binding assays, we demonstrated that this binding occurs via the β′- and γ-COP subunits of coatomer (17,18). All these data combined functionally tie in Glo3p tightly with the COP I pathway.

Surprisingly, in mammalian cells only ARFGAP1, which by sequence analysis can be clearly identified as a Gcs1p orthologue, has been well characterized so far. ARFGAP was the first ARF-directed GAP to be discovered (19). ARFGAP1 shuttles between the Golgi complex and cytosol and is involved in COP-I-dependent trafficking *in vivo*(20,21). Assays with Golgi membranes and recombinant proteins *in vitro* have suggested that ARFGAP1 is required for COP I vesicle generation, and under certain *in vitro* conditions ARFGAP1 can be observed as a stoichiometric component of the COP I coat, namely in vesicles produced in the presence of GTP rather than GTP-γ-S (22,23).

Significantly, however, a *Drosophila* mutant that lacks the fly orthologue of ARFGAP1 encoded by the *Gap69C* gene is viable and fertile (24), consistent with the notion that it is not an essential, key component to COP I function and that other, presumably Glo3p-type ARFGAPs can compensate for the loss of ARFGAP1 function in the fly. In this study, we aimed to identify and characterize human Glo3p orthologues.

Sixteen genes encoding ARFGAPs are found in the human genome. Using a conserved signature motif present in GLO3 proteins from all species, named the Glo3 motif, we unambiguously identify ARFGAP2 and ARFGAP3 as the two human orthologues of yeast Glo3p, and we provide their initial cell biological characterization. ARFGAP1, ARFGAP2 and ARFGAP3 are coexpressed in mammalian cells (e.g. HeLa and NRK cells). Our data strongly suggest that ARFGAP1, ARFGAP2 and ARFGAP3 co-operate to perform essential, overlapping functions in COP-I-mediated trafficking in mammalian cells.

## Results

### Two human orthologues of yeast Glo3p

We wished to identify a human orthologue for the yeast ARFGAP Glo3p, which in yeast has been demonstrated to have a prominent role in coatomer-mediated trafficking. Among over 16 human proteins with an ARFGAP domain, we found human Gcs1p, which is named ARFGAP1, and four novel proteins with a closely related ARFGAP domain. In order to identify a true orthologue of yeast Glo3p, we searched for proteins with sequence similarity beyond the ARFGAP domain. Making use of a Glo3 protein from *Schizosaccharomyces pombe*, we were able to identify a small conserved signature motif, named the Glo3 motif, found in 19 ARFGAP proteins from 14 different species. Using this information, we found two distinct human orthologues of yeast Glo3p, ARFGAP2 and ARFGAP3. In summary, searching the increasing amount of data available from systematic sequencing projects, we identified two distinct human sequence orthologues of the yeast ARFGAP Glo3p.

### The zinc finger domain and the Glo3 motif

**Figure 1:**
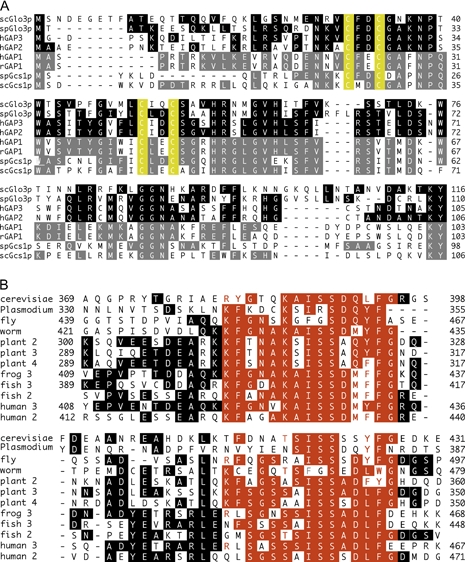
**ARFGAP domain and conserved motif of a representative subset of Glo3 proteins.** A) The N-terminal ARFGAP domains of Glo3 and Gcs1 proteins from *Saccharomyces cerevisiae*, *Schizosaccharomyces pombe* and *Homo sapiens* were aligned using the clustal V algorithm in the MegAlign program (DNAStar package). Note the conserved cysteine residues highlighted in yellow and the overall high degree of sequence conservation in this domain. B). The Glo3 motif (highlighted in orange) consists of a repeat of 15 residues separated by 11–17 residues. It is a signature motif that allows unambiguous identification of Glo3 orthologues. Genbank accession numbers: cerevisiae: 6320969 (*S.* *cerevisiae*); Plasmodium 23478251 (*P.* *yoelii*); worm: 25153991 (*Caenorhabditis elegans*); fly: 24668642 (*Drosophila melanogaster*); plant A: 7487780, plant B: 18403775, plant C 15237500 (*Arabidopsis thaliana*); fish: assembled from several ESTs: 12148139, 12171549, 12158629 and 12265392 (*Danio rerio*); frog: 27695479 (*Xenopus laevis*) and human A: 21263420 and human B: 31543983 (*H.* *sapiens*).

Both Glo3p and Gcs1p share high sequence homology in their amino terminal catalytic zinc finger domain, which is known to engage ARF. Figure 1A shows an alignment of Glo3p, Gcs1p and their orthologues from *S.* *pombe*, as well as rat and human ARFGAP1, and the human ARFGAP2 and ARFGAP3. Note the presence of the four conserved cysteine residues characteristic for the zinc finger domain of ARFGAPs highlighted in yellow.

The conserved Glo3 motif, which is characteristic for Glo3 proteins from a wide variety of eukaryotic organisms including plants, is about 45 amino acid residues in length and is situated in the carboxy terminal part of the Glo3 proteins (see Figure 1B). It consists of two repeats of 15 residues separated by a linker of 19–23 residues, depending on the species. Gaps immediately after some of the repeats suggest flexibility in the exact length of the sequence in the region (Figure 1B). Not surprisingly, the overall degree of conservation is lowest in the parasitic amoeba *Plasmodium yoelii* and the fruit fly *Drosophila melanogaster*. In these organisms, only a single Ile-Ser-Ile tripeptide is present in the second repeat, whereas only the first repeat is well conserved in the nematode *Caenorhabditis elegans*. During preparation of this manuscript, Nakano’s group also noted the presence of the Glo3 motif and could demonstrate that it is important for Glo3p function in yeast (16).

**Figure 2:**
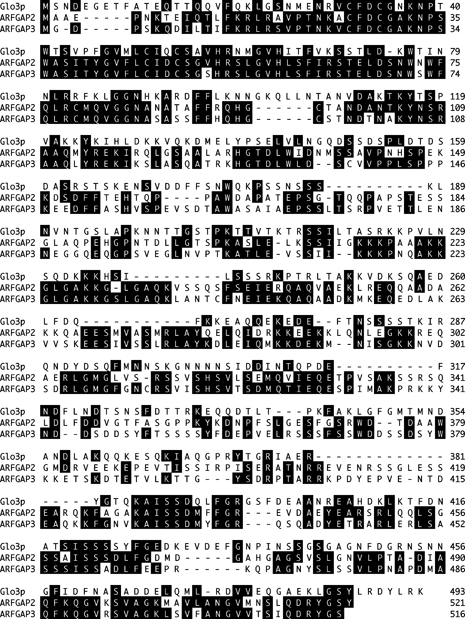
**Sequence alignment of yeast Glo3p and human ARFGAP2 and ARFGAP3.** ARFGAP2 and ARFGAP3 share 49.6% protein sequence identity. ARFGAP2 and ARFGAP3 share 17.6 and 19.9% sequence identity with Glo3p, respectively.

The Glo3 motif, which is always found strictly associated with an ARFGAP domain, allowed us to identify the true human orthologues of yeast Glo3p as ARFGAP2 and ARFGAP3, by sequence comparison. In Figure 2, a protein sequence alignment of yeast Glo3p, ARFGAP2 and ARFGAP3 is shown. ARFGAP2 is a protein of 521 residues and had not been previously named. However, a zinc finger protein of unknown function and designated Zfp289 had been previously identified from SCp2 mouse mammary epithelial cells (25). The protein sequence of human ARFGAP2 and mouse Zfp289 shows complete identity. ARFGAP2 and ARFGAP3 share 49.6% protein sequence identity. In an alignment over their entire length, Glo3p shares only 17.6 and 19.9% identity with ARFGAP2 and ARFGAP3, respectively. As for ARFGAP3 (516 residues), both the presence of the ARFGAP domain and its function as a GTPase-activating protein towards ARF *in vitro* had been noted (26,27). However, neither ARFGAP2 nor ARFGAP3 have previously been recognized as Glo3p rather than Gcs1p orthologues, and the localization of both ARFGAP2 and ARFGAP3 and their role in COP I transport has not been investigated.

### ARFGAP2 and ARFGAP3 are found in the Golgi complex as well as in pre-Golgi punctate structures

**Figure 3:**
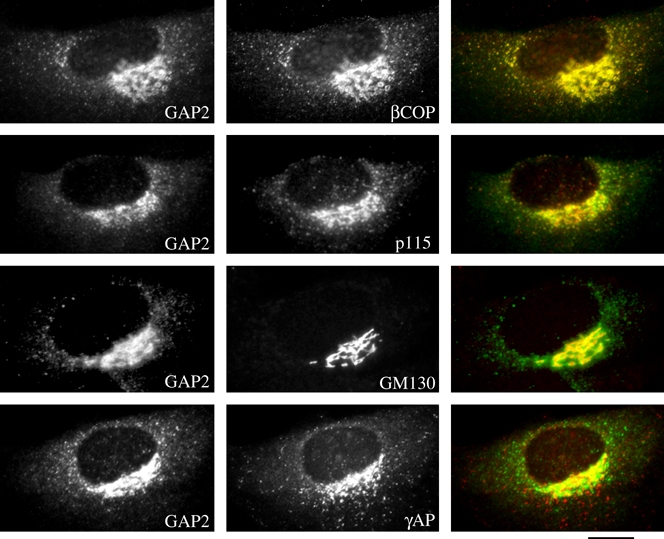
**ARFGAP2 colocalizes with coatomer in NRK cells.** Methanol–acetone-fixed NRK cells were stained with affinity-purified ARFGAP2 antibodies and monoclonal antibodies against β-COP, p115, GM130 or γ-adaptin. Cy3-coupled anti-rabbit and Alexa488-coupled anti-mouse secondary antibodies were used. Images were acquired using a Zeiss Axioplan Fluorescent Microscope equipped with a charge-coupled device camera. The individual channels are shown in monochrome; the composite overlay is shown to the right (green channel, ARFGAP2; red channel, β-COP, p115, GM130 and γAP, respectively). Bar = 10 μm.

**Figure 4:**
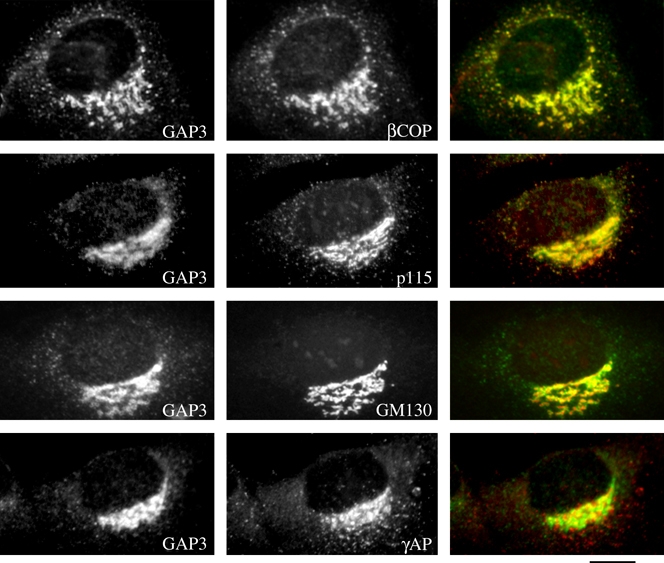
**ARFGAP3 colocalizes with coatomer.** Methanol–acetone fixed NRK cells were labelled with affinity-purified ARFGAP3 antibodies and mAbs against β-COP, p115, GM130 or γ-adaptin (see Figure 3 for other details). The individual channels are shown in monochrome; the composite overlay is shown to the right (green channel, ARFGAP3; red channel, β-COP, p115, GM130 and γAP, respectively). Bar = 10 μm.

In order to confirm that these two human Glo3p orthologues are involved in COP I transport, we raised monospecific polyclonal antibodies against them (see *Materials and Methods*) and determined their intracellular localization. By IF studies on methanol–acetone-fixed NRK cells, we find the majority of ARFGAP2 as well as ARFGAP3 associated with a juxtanuclear densely packed structure typical for the mammalian Golgi complex (Figures 3 and 4). The remaining protein is associated with small punctate structures scattered throughout the cytoplasm. Double-labelling experiments reveal that these structures labelled with antibodies against ARFGAP2 as well as ARFGAP3 are largely identical to those labelled with antibodies against subunits of the coatomer complex, identifying them as ER-Golgi intermediate compartment (ERGIC)/vesicular-tubular compartment (VTC) structures.

To extend these results, we performed double-labelling experiments with a number of well-established marker proteins. The tethering protein p115 is known to be associated with the early Golgi complex as well as with a punctate pre-Golgi compartment (28). Although because of its exceptional quality, the monoclonal antibody against p115 labels a considerably larger number of peripheral structures, the majority of ARFGAP2- or ARFGAP3-positive structures are p115 positive as well. Within the Golgi compartment, there are areas where closer inspection of the patterns suggests that the mammalian ARFGAP2 and ARFGAP3, and p115, respectively, are localized in a closely associated but not identical pattern. This is in clear contrast to these proteins in comparison with subunits of the coatomer complex, where most features within the Golgi complex are labelled in a remarkably identical way.

Again, this is not the case for ARFGAP2 and ARFGAP3 when compared with the well-characterized early Golgi marker GM130 (29) (see Figures 3 and 4). In this case, the proteins are localized in a closely associated but not identical fashion in the region of the Golgi complex. As a control, we analysed the localization of ARFGAP2 and ARFGAP3 in comparison to γ-adaptin, asubunit of the clathrin adaptor complex AP-1, which is involved in traffic between the *trans*Golgi network (TGN) and endosomal compartments (30). As expected, we found no significant overlap in the extensive punctate peripheral label as well as the juxtanuclear label associated with the Golgi complex (Figures 3 and 4). We conclude that the localization of ARFGAP2 and ARFGAP3 is consistent with a function in COP I trafficking.

### ARFGAP2 and ARFGAP3 are associated with COP-I-coated vesicles generated *in vitro*

**Figure 5:**
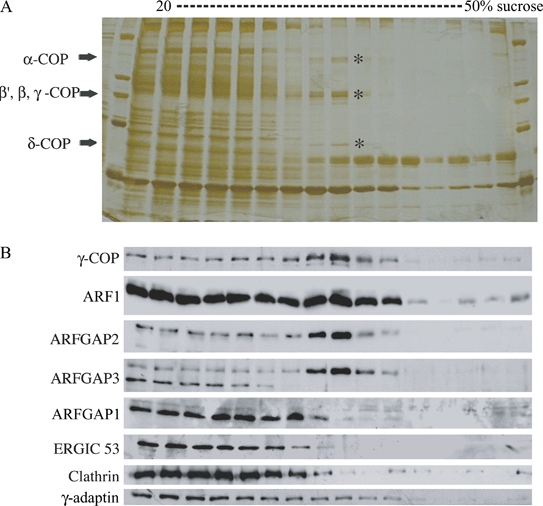
**ARFGAP2 and ARFGAP3 are associated with COP I vesicles generated *in vitro*.** Vesicle budding reactions were performed according to the protocol developed by (31), using the non-hydrolysable GTP analogue, GTP-γ-S. Vesicles produced in the reaction were separated from the Golgi donor membranes on sucrose density gradients and peaked at 40–43% sucrose. Fractions were analysed by silver stain (upper panel) and immunoblotting with antibodies as indicated (lower panel). The positions of coatomer bands are indicated on the left and also by asterisks. Note the abundant presence of ARFGAP2 and ARFGAP3 in the vesicle fractions defined by the presence of coatomer subunits (fractions 8 + 9) but the absence of ARFGAP1.

We next wished to test whether ARFGAP2 and ARFGAP3 are associated with COP I vesicles produced *in vitro*. For this we employed the ‘classic’ budding assay developed by the Rothman/Wieland labs (31,32), using purified rat liver Golgi and pig brain cytosol. The budding reaction was performed in the presence of the nonhydrolysable GTP analogue, GTP-γ-S, which locks ARF on the membrane of vesicles and thus prevents uncoating. Vesicles and Golgi donor membranes were separated on a linear sucrose gradient by overnight centrifugation (see *Materials and Methods*, and 31), and proteins in the fractions obtained were analysed by immunoblotting and silver staining of bands after SDS–PAGE (Figure 5). The characteristic set of coatomer bands (α-, β’-, β-, γ- and δ-COP) were found enriched in the expected positions for COP-I-coated vesicles in the gradient (fractions 8 + 9; corresponding to 40–43% sucrose) where also the blot signals for γ-COP (Figure 5A,B) and β-COP (data not shown) showed a corresponding major peak. ARFGAP1 was predominantly detected in the donor Golgi fractions, but only small amounts were found in the fractions containing COP I vesicles (Figure 5B). This is consistent with the previously reported findings from the Hsu lab that ARFGAP1 is depleted from COP I vesicles formed in the presence of GTP-γ-S (23). On the other hand, ARFGAP2 and ARFGAP3 showed a strong peak in the COP I vesicle fractions (Figure 5B), indicating that these novel Glo3-type ARFGAPs can be actively recruited into budding COP I vesicles even in the presence of GTP-γ-S. Clathrin heavy chain and the γ-subunit of the AP-1 adaptor complex used as controls were absent from the COP I vesicle fractions, as expected. We find that the dilysine motif-bearing protein ERGIC-53 was also not included into the COP I vesicles. ADP-ribosylation factor-1, which is involved in many different transport steps within the Golgi complex, was found both in the donor Golgi fractions and in the COP I vesicle fractions, as expected. Our data show that both novel ARFGAPs, ARFGAP2 and ARFGAP3 are associated with the COP-I-coated vesicles produced *in vitro* in the presence of GTPγS, whereas ARFGAP1 is not or much less so.

### The catalytic domain on Glo3p or ARFGAP2 is not required for interaction with coatomer

**Figure 6:**
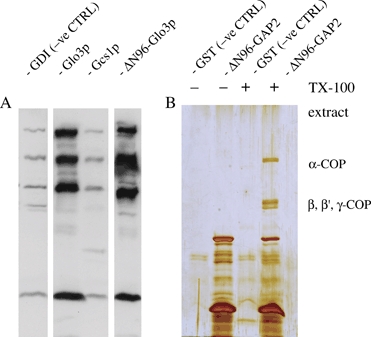
**Direct interaction of yeast and human Glo3 with coatomer.** His6- or GST-tagged proteins were used to pull down coatomer from yeast or rat liver cytosol, under conditions described by us previously (17). A) His6-tagged Glo3p lacking its amino terminus was compared with full-length Glo3p as well as full-length Gcs1p in its ability to bind coatomer from yeast cytosol. Protein bound to Ni-NTA agarose beads was resolved by SDS–PAGE, followed by Western blotting and incubation with anti-yeast coatomer antibodies detected by ECL. His-tagged Glo3p lacking its ARFGAP domain (ΔN96) still binds coatomer from yeast cytosol *in vitro*, whereas Gcs1p does not. GTP dissociation inhibitor is used as a negative control here. B) A GST-fusion protein harbouring ΔN96-ARFGAP2 was used for a pull-down from a centrifugation-cleared TX-100 extract of pig brain crude microsomal membranes. Glutathione S-transferase was used as the negative control. Note absence of binding in the negative control and the presence of the characteristic coatomer bands in the pull-down involving the ΔN96-ARFGAP2 GST fusion as the fishing hook. Enrichment of α- and β-COP in the bound fraction was confirmed using antipeptide antibodies (data not shown).

Yeast Glo3p interacts with γ-COP as well as β′-COP in the two-hybrid system (17). This strong, direct interaction was confirmed by pull-down experiments using recombinant His-tagged Glo3p and yeast cytosol (17). Here we show that the catalytic domain of yeast Glo3p is not required for this direct interaction with coatomer. Deletion of the N-terminal 96 amino acids including the Zn finger domain does not reduce the interaction of tagged Glo3p with coatomer *in vitro* (Figure 6A). In this type of experiment, Gcs1p does not bind coatomer above background levels defined by recombinant GTP dissociation inhibitor (GDI) as a negative control (Figure 6A). In order to test whether mammalian ARFGAP2 interacts with coatomer in a way comparable to yeast Glo3p, glutathione S-transferase (GST)-tagged ARFGAP2 lacking its ARFGAP domain was used for pull-down experiments from rat liver cytosol. Indeed, coatomer binding from rat liver cytosol to GST-tagged ARFGAP2 present on glutathione beads was readily detectable by silver stain (Figure 6B), and immunoblots using anti-peptide antibodies against α- and β-COP demonstrated enrichment of these coatomer subunits (data not shown). Thus, the catalytic domain of Glo3-type ARFGAP proteins from yeast and mammals is not required for *in vitro* interaction with coatomer.

### Expression of ΔN-ARFGAP2–cyan fluorescent protein inhibits CTX transport

**Figure 7:**
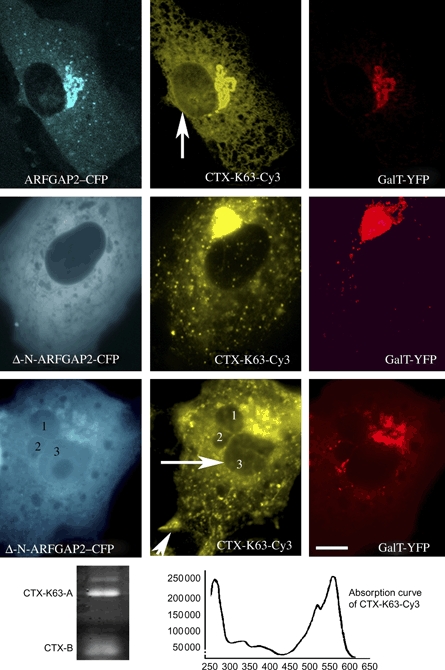
**ARFGAP2 is involved in COP-I-dependent trafficking in Vero cells.** Vero cells coexpressing full-length ARFGAP2–CFP and the Golgi marker GalT-YFP were treated with CTX-K63-Cy3 (upper panel). After 3 h of internalization, CTX-K63-Cy3, the Cy3-labelled A-subunit, is prominently present in fine ER structures (including the nuclear envelope; arrow) and in the Golgi complex. ARFGAP2–CFP colocalizes well with GalT-YFP in the Golgi. Additionally, ARFGAP2–CFP is present in scattered punctate structures, most likely intermediate compartment. In contrast, in Vero cells expressing ΔN-ARFGAP2–CFP2 transport of CTX-K63-Cy3 to the ER is inhibited (middle panel). ΔN-ARFGAP2–CFP2 localizes to the Golgi complex. Vero cells overexpressing ΔN-ARFGAP2–CFP2 for extended periods often display two to three nuclei and show severe inhibition of toxin transport (lower panel). The bottom panel is the characterization of the Cy3-labelled CTX-K63 (non-toxic AB5 holotoxin) used for the experiments (for details see methods). Mostly the A-subunit of CTX-K63 is labelled, with traces of B present. Scale bar: 10 μm.

Full-length ARFGAP2 was tagged at the carboxy terminus with cyan fluorescent protein (CFP) and expressed at low level in Vero cells. This CFP fusion protein localized to the Golgi complex [as identified using the Golgi enzyme marker galactosyl transferase tagged with yellow fluorescent protein (GalT-YFP)] and also localized to punctate structures scattered through the cytoplasm (Figure 7, upper panel). This localization pattern is reminiscent of the localization observed with antibodies against ARFGAP2 (compare with Figure 3).

To assay COP-I-dependent transport *in vivo*, we employed a model cargo protein previously described by us (21,33), the non-toxic mutant version of cholera toxin fluorescently labelled with the dye Cy3. After 3 h of internalization, CTX-K63-Cy3 had been endocytosed from the plasma membrane and was prominently present in the Golgi complex and in the ER network (Figure 7, upper panel). In cells expressing higher levels of ARFGAP2–CFP, a strong cytoplasmic staining appeared in addition to the Golgi pattern, perhaps suggesting a limited number of binding sites on the Golgi. Importantly, however, transport of CTX-K63-Cy3 still proceeded with kinetics very similar to control cells (data not shown).

To test whether ARFGAP2 may be involved in COP-I-dependent transport *in vivo*, we generated a ΔN-ARFGAP2–CFP mutant. At low expression level, this protein was detected at the Golgi complex when expressed in Vero cells (not shown). At elevated expression levels (12 h after transfection), a strong cytoplasmic pattern was seen in addition to the Golgi pattern (Figure 7, middle panel). Importantly, in such cells, 3 h after addition, CTX-K63-Cy3 was not observed in the ER but remained restricted to the Golgi complex and punctate structures scattered through the cytoplasm (Figure 7, middle panel). These data indicate that expression of ΔN-ARFGAP2–CFP interferes with COP-I-dependent transport of CTX-K63-Cy3 to the ER.

**Figure 8:**
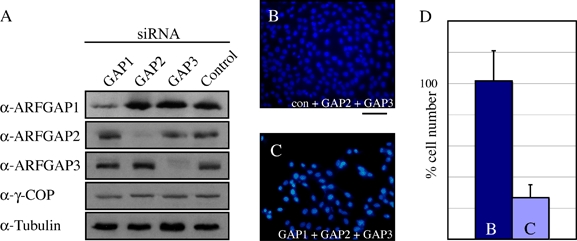
**Silencing of all three ARFGAPs is lethal in HeLa cells.** Silencing of ARFGAP1 or a combination of ARFGAP2 and ARFGAP3 failed to cause lethality in HeLa cells, as well as in NRK cells (data not shown). A) The depletion of ARFGAP1, ARFGAP2 or ARFGAP3 in single knock-downs in HeLa cells was verified by immunoblotting. Antibodies against tubulin and γ-COP were used to verify equal protein loading. B and C) Cell counting was performed from DAPI-stained HeLa cells grown on coverslips. Note the strong reduction in cell numbers in the ARFGAP triple knock-down compared with a double knock-down involving ARFGAP2 and ARFGAP3 combined with a control oligo. For details of the oligos, see *Materials and Methods*. D) Quantification of the reduction in cell numbers from the above. More than 75% cell death was routinely observed after three sets of siRNA transfections at the 72-h time-point after the last transfection (in 10 independent experiments). Bar = 100 μm.

At even longer time-points after transfection (16 h), cells expressing ΔN-ARFGAP2–CFP displayed a strikingly higher frequency of having two or even three nuclei (indicated by numbers in Figure 8, lower panel). In such cells, as judged by GalT-YFP, usually only one large Golgi complex was present to which ΔN-ARFGAP2–CFP localized (Figure 7, lower panel). In such cells, transport of CTX-K63-Cy3 was again severely inhibited, with the toxin not arriving at the ER network even after 3 h. Instead, CTX-K63-Cy3 was present in the Golgi complex and large cytoplasmic structures (Figure 7, lower panel). Our data strongly suggest that ARFGAP2 is involved in the COP-I-dependent trafficking of cholera toxin from the Golgi to the ER.

### Silencing of all three ARFGAPs is lethal

In order to further investigate the role of ARFGAP2 and ARFGAP3 in COP-I-mediated transport, we analysed NRK and HeLa cells upon knock-down of individual ARFGAP transcripts. Protein levels were monitored by immunoblots of total cell extracts from NRK cells 72 h after transfection with appropriate small interfering RNA oligonucleotides (siRNAs). As shown in Figure 8, we indeed found oligos that allowed knock-down of the individual ARFGAPs in transfections with a single RNAi oligo. Antibodies against γ-COP and β-tubulin were used to confirm equal loading of the samples. In yeast, deletion of either the GLO3 or GCS1 gene results in a relatively mild phenotype. However, deletion of both genes is lethal. Therefore, we tested the effect on cell survival of an RNAi-oligo-mediated knock-down of ARFGAP1, ARFGAP2 and ARFGAP3 either in pairs or as a combined triple knock-down (see *Materials and Methods* for details). No significant cell death was observed in single knock-downs or in any pairwise knock-down, compared with cells transfected with a control oligo. However, a triple knock-down of ARFGAP1, ARFGAP2 and ARFGAP3 in HeLa cells resulted in strongly significant cell death. More than 75% cell death was routinely observed after three sets of siRNA transfections at the 72-h time-point after the last transfection. Very similar results were observed in NRK cells (data not shown). We conclude that at least one of the three ARFGAPs is required to maintain cell viability.

## Discussion

We wish to understand the role of ARFGAPs in the regulation of COP-I-dependent trafficking. In yeast, the two ARFGAPs Glo3p and Gcs1p together provide an overlapping, essential function in COP-I-dependent membrane traffic. In mammalian cells, only the Gcs1p orthologue, ARFGAP1 has been characterized so far. In this study, we provide an initial cell biological characterization of two recently identified human ARFGAPs, ARFGAP2 and ARFGAP3. We unambiguously demonstrate by sequence analysis that they are the human orthologues of yeast Glo3p, through the presence of a conserved ‘signature’ motif at the carboxy terminus, the Glo3 motif. Using novel antibodies, we demonstrate that they are localized to the Golgi complex and peripheral punctate structures scattered throughout the cytoplasm in NRK cells. Both ARFGAP2 and ARFGAP3 significantly colocalize with COP I subunits in both locations, strongly suggesting that they are associated with COP I on both ERGIC/VTC structures and at the Golgi complex. Here we further show that both ARFGAP2 and ARFGAP3 are associated with COP I vesicles produced *in vitro* in the presence of the non-hydrolysable GTP analogue, GTP-γ-S.

The Golgi and VTC localization of ARFGAP2 and ARFGAP3 appears to be dynamic, as treatment with brefeldin A (BFA) rapidly renders their localization completely diffuse-cytoplasmic within 2–5 min of adding the drug, with kinetics very similar to those of β-COP and ARFGAP1 (unpublished data). Similarly, re-recruitment of ARFGAP2 and ARFGAP3 to Golgi membranes occurs rapidly upon washout of the drug. Future experiments will need to address the dynamics of ARFGAP2/3 using live cell imaging approaches, similar to detailed analysis that has been reported for ARFGAP1 (20).

The Nakano group has recently reported that Glo3p but not Gcs1p can suppress arf-Ts mutants with a severe defect in Golgi-to-ER retrieval in an allele-specific manner (16). They found that the Glo3 motif is required for suppression of the growth defect of *arf1-16* and *arf1-17*, suggesting that the motif is important for Glo3p function (16).

We had previously shown that ARFGAP2, and more weakly ARFGAP3, can interact with recombinant carboxy terminal γ-COP ‘ear’ domain in pull-down experiments from rat liver cytosol (18). Furthermore, we could show this interaction is likely to be physiologically important, as plasmid-driven overexpression of the γ-COP ‘ear’ domain in NRK cells disrupted the Golgi localization of ARFGAP2, whereas a point mutant version of the γ-COP ‘ear’ domain (W776S) in which the binding site to ARFGAP2 is abrogated had no effect (18). Here we show that the catalytic domain of ARFGAP2 and ARFGAP3 is not required for *in vitro* interactions with mammalian coatomer. The same phenomenon was observed for their yeast counterpart Glo3p in binding experiments using yeast cytosol. It has recently been shown that ARFGAP1 can also directly interact, albeit more weakly, with coatomer. However, this interaction involves two distinct binding sites on ARFGAP1, one in its catalytic domain and the other in the non-catalytic region (see 23). Using the two-hybrid system, we were unable to detect interactions of Gcs1 or ARFGAP1 with yeast or mammalian coatomer subunits, respectively, whereas we observed strong interactions of Glo3p as well as ARFGAP2 and ARFGAP3 with coatomer subunits (17, unpublished data). We show that in the presence of GTP-γ-S, ARFGAP2 and ARFGAP3 are able to associate with COP-I-coated vesicles whereas ARFGAP1 is barely detectable.

In most mammalian cells there are six ARFs, of which ARF1 is the most extensively studied. It has been implicated in Golgi-to-ER transport, function of the Golgi, transport from the trans-Golgi network, transport in the endocytic pathway and recruitment of paxillin to focal adhesions (7). Because in mammalian genomes the number of proteins with an ARFGAP domain is considerably larger than the number of ARF proteins, it has been suggested that a number of GAPs might regulate the activities of ARF1 in a location-dependent manner.

The observed interaction of Glo3p-type ARFGAPs, namely Glo3p in yeast and ARFGAP2 and ARFGAP3 in mammals, with coatomer subunits (17,18; this study) may thus enable regulation of aspects of the COP I vesicle cycle, such as cargo sorting, coat formation, membrane deformation or uncoating, which may enable regulation of traffic at different intracellular sites and at specific intracellular sites. It is perhaps surprising, given the strong direct interactions of ARFGAP2 and ARFGAP3 with coatomer, that these proteins were not previously identified in purified COP I vesicle populations. Apparently, in isolated COP-I-coated vesicles produced in the presence of GTP-γ-S (31; this study), neither ARFGAP2 nor ARFGAP3 are stoichiometric coat components.

There are precedents for specific interactions of ARFGAPs with other coat proteins. For example, it has been shown that AGAP1 can directly interact with the Golgi/endosome-localized AP-3 adaptor but not with the closely related AP-2 adaptor involved in endocytosis from the plasma membrane (34,35). The related AGAP2 interacts specifically with the AP-1 adaptor to regulate trafficking from recycling endosomes (35) but not with AP-2. The functional significance of the direct interactions of ARFGAP2 and ARFGAP3 with coatomer with regard to protein sorting into COP I vesicles and the regulation of uncoating once the vesicles are fully assembled needs to be addressed in future experiments.

In yeast, Glo3p and Gcs1p provide an essential, overlapping function for COP-I-mediated transport (12). However, growing evidence suggests that while Gcs1p is able to maintain ARF1-regulated membrane traffic in several transport steps, Age2p and Glo3p are specialized for a transport step out of the TGN (36) and COP-I-mediated transport, respectively (12). Our *in vivo* data demonstrate that ARFGAP2 is involved in the COP-I-dependent Golgi-to-ER transport of a model cargo, cholera toxin.

We show here that a combined knock-down of ARFGAP1, ARFGAP2 and ARFGAP3 using siRNAs is lethal in HeLa and NRK cells. Thus, our data strongly suggest that, similar to the situation yeast, Gcs1p-type and Glo3p-type ARFGAPs together provide an essential function for COP-I-mediated transport. In the future, it will be important to analyse in detail the cellular phenotypes resulting from RNAi-mediated gene silencing of individual ARFGAPs and use *in vivo* and *in vitro* assays to unravel the individual contributions of ARFGAP1, ARFGAP2 and ARFGAP3 to COP-I-mediated membrane traffic.

## Materials and Methods

### DNA cloning, sequencing and computer analysis

*Escherichia coli* strain DH5α was used for plasmid isolation, and polymerase chain reaction reactions using a combination of AmpliTaq and TaqExtender or Vent DNA polymerase, restriction enzyme digests and ligations were performed by standard methods. All constructs were verified by DNA sequencing. Database searches were performed using the blast and Ψblast servers at National Institutes of Health (NIH). Multiple alignments were performed with the program MegAlign using the clustal V algorithm.

### Plasmids and image clones

Full-length clones for both human Glo3 proteins were obtained from the image consortium. A hypothetical intron–exon structure established for the ARFGAP2 locus on chromosome II allowed to explain a number of splice variants and artefacts found in the expressed sequence tag (EST) database. Splice variants most closely related to yeast Glo3p were chosen for further experiments: image clone 650′690 for ARFGAP3 and a combination of image clone 2′961′498 (ATG to NcoI site) and clone 2′986′241 (NcoI to TGA) for ARFGAP2. The coding region of several image clones with full-length inserts were sequenced.

### Cloning of ARFGAP2 and ARFGAP3, expression plasmids and raising antibodies

To obtain His6-tagged recombinant proteins as antigen and for binding experiments, DNAs encoding full-length ARFGAP2 and ARFGAP3 were cloned into pET21d (Novagen) and expressed in λDE3 lysogens of strain BL21 and purified by Ni-NTA chromatography as described (17). Purified His6-tagged ARFGAP2 and ARFGAP3 were used for raising antibodies in rabbits. Antibodies were affinity purified over immobilized GST-tagged ARFGAP2 and ARFGAP3 protein lacking the ARFGAP domain to reduce the risk of cross-reactivity of these antibodies between the two proteins and characterized by immunoblotting and IF. Immunoblots on total protein extracts from NRK and HeLa cells revealed that our antibodies recognized the human and rat proteins with high affinity, that there is a small difference between the two proteins in their apparent molecular weight in SDS-polyacrylamide gels and that both proteins are expressed at readily detectable levels (data not shown).

### Cell lines and commercial antibodies

HeLa cells, NRK and Vero (green monkey kidney fibroblast) cells were maintained in DMEM medium + 10% bovine calf serum, 2 mm glutamine and antibiotics penicillin and streptomycin (100 U/mL and 100 μg/mL, respectively). In some experiments, BFA was added to the cell culture medium at a concentration of 5 μg/mL. A stock solution of BFA (2 mg/mL in ethanol; Epicentre Technologies) was stored at −20°C.

For colocalization analysis by IF, we used monoclonal antibodies against GM130 (clone NN 2C10; Abcam 1299), p115 (clone 5D6; Sigma P3118), γ-adaptin (clone 100/3; Sigma4200) and β-COP (clone mAD; Abcam 6323).

### IF and confocal fluorescence microscopy

NRK or HeLa cells grown on glass coverslips were fixed in methanol at −20°C for 4 min followed by fixation for 30 seconds with −20°C acetone. Cells were mounted in 50% glycerol on glass slides and epifluorescence microscopy was performed on a Zeiss Axioplan microscope with a 63×, 1.4 oil immersion objective, with images taken using a CoolSnap CCD camera.

### Pull-down experiments and immunoblot analysis

His6- or GST-tagged proteins were used to pull down coatomer from yeast or rat liver cytosol, under conditions described by us previously (17). His6-tagged Glo3p lacking its amino terminus was compared with full-length Glo3p as well as full-length Gcs1p in its ability to bind coatomer from yeast cytosol. Protein bound to Ni-NTA agarose beads was resolved by SDS–PAGE, followed by Western blotting and incubation with anti-yeast coatomer antibodies detected by enhanced chemiluminescence (ECL). A recombinant GST-fusion protein harbouring ΔN96-ARFGAP2 was used for pull-downs from rat liver cytosol. Glutathione S-transferase alone or recombinant GST-tagged GDI (a guanine nucleotide dissociation inhibitor acting on rab proteins) were used as negative controls. SDS–PAGE and immunoblotting analysis using ECL were performed as described (17).

### *In vitro* budding reactions

*In vitro* budding of COP-I Golgi-derived vesicles from purified rat liver Golgi membranes was performed in the presence of pig brain cytosol as a source of coatomer, ATP and the nonhydrolysable GTP analogue, GTP-γ-S as described by the Rothman lab (31,32). Pig brain cytosol was prepared by the method as described by (37). Donor Golgi membranes were prepared according to a protocol established by the Graham Warren lab (38). Vesicles were separated from Golgi donor membranes by sedimentation to equilibrium in a sucrose density gradient. Following trichloroacetic acid precipitation and SDS–PAGE on 10% gels, proteins in fractions were analysed by silver stain and ECL immunoblot using specific antibodies as indicated.

### Cell transfections and application of cholera toxin CTX-K63 and CFP- and YFP-fusion proteins

Vero cells were transfected by electroporation as described by us earlier (21). Unless otherwise mentioned, cells were tested for expression of fluorescent fusion proteins and cholera toxin binding 6 h after transfection. For experiments, cells were treated in a pulse-like manner with CTX-K63, as described before (21,33). CTX-K63 is a nontoxic mutant of cholera toxin, which has no ADP-ribosylating activity as a result of a Ser_63_/Lys_63_ point mutation in the A-subunit (21,33). Following treatment with CTX-K63, cells remained in the CO_2_ incubator until they were transferred to the thermostated microscope chamber. Internalization of CTX-K63-Cy3, labelled as described below, was performed for 3 h at 37°C before imaging.

An expression clone for an YFP-tagged version of the Golgi-resident enzyme galactosyl transferase (GalT-YFP) was a kind gift from J. Lippincott-Schwartz (NIH). We constructed CFP-tagged full-length or ΔN96-ARFGAP2 expression clones, with the CFP moiety at the carboxy terminus of the proteins, using standard cloning procedures in the vector pECFP-C1 (ClonTech).

### Cy3 labelling of CTX-K63

As described by us (21), CTX-K63 mutant toxin was labelled with Cy3, resolved by 10% SDS–PAGE and scanned using a Typhoon 8600 Imager with an excitation wavelength of 532 nm and standard emission filter 560LP. Pixel-by-pixel resolved fluorescence measurement revealed preferential labelling of the A-subunit in the sample that was used in the experiments described in Figure 7. The absorption spectrum of the CTX-K63-Cy3 sample was acquired with a Fluoromax-3 spectrofluorimeter. The wavelength ratio of 550 nm/280 nm, that is, of dye (550 nm) to protein (280 nm), was used to measure the concentration of proteins in the sample and to estimate the labelling ratio, calculated using Beer’s law and the dye extinction coefficient of Cy3 150 000 M^−1^ cm^−1^. From this presence of approximately 1.5–2 dye molecules per A-subunit were determined.

### siRNA

Sequences were designed following the parameters set out by Dharmacon by analysing the human genomic sequence for each protein. The following sequences were selected: ARFGAP1, AAG GUG GUC GCU CUG GCC GAA G (siACE-RNAi OPTION C DUPLEX); ARFGAP2, AAG CUA UGG GGU GUU UCU CUG (siACE-RNAi OPTION C DUPLEX); and ARFGAP3, AAC CUA UGG AGU GUU CCU UUG (siACE-RNA OPTION C DUPLEX). All oligos were custom synthesized by Dharmacon (http://www.Dharmacon.com/). A green fluorescent protein duplex was used as a control oligo for all experiments (Dharmacon, D-001300-01-20).

### RNAi-mediated knock-downs of ARFGAP1, 2 and 3 in HeLa cells

HeLa M cells were maintained in DMEM medium supplemented with 10% foetal bovine serum, 2 mml-glutamine, penicillin (100 U/mL) and streptomycin (100 μg/mL). siRNAs were used at a final concentration of 20 nm in all transfection experiments. Triple knock-down of all three ARFGAPs and combinations of these with controls were performed in a staggered 10-day protocol, using oligofectamine. For double transfections, paired siRNA oligos were added to 15 nm final concentrations. Cells were seeded on day 0 and transfected on day 1 with either control, single ARFGAP or ARFGAP2 and 3 oligos. On day 2, cells were split into new wells, allowed to adhere to the dish for 6 h, then transfected with either control or ARFGAP1 oligo. On day 3, the media was changed to remove transfection reagents. On day 5, cells were split, allowed to adhere for 6 h and transfected as on day 1. On day 6, the media were refreshed, and on day 7, cells were transfected as on day 2. On day 8, cells were split (some cells were reseeded onto coverslips for 4′-6′-diamidino-2-phenylindole (DAPI) staining. On day 9, the cells rested, and on day 10, cells were collected for analysis. For immunoblot samples, cells washed with PBS, then lysed directly in the plate by addition of 3× Laemmli sample buffer, pipetted out and immediately boiled at 95°C for 5 min. DNA was sheared by passing the samples through a narrow gauge needle and vortexing at maximum speed. Samples were then loaded onto SDS–PAGE gels. Cells growing on coverslips for DAPI staining were washed three times in PBS, then methanol–acetone fixed and mounted on to glass slides in DAPI-containing mounting medium (10 μg/mL DAPI, 1 mg/mL p-phenylenediamine in 90% glycerol, 10% PBS, pH 8.0). A similar protocol was used for NRK cells, yielding almost identical results.
